# Promotion of mitochondrial fusion protects against developmental PBDE-47 neurotoxicity by restoring mitochondrial homeostasis and suppressing excessive apoptosis

**DOI:** 10.7150/thno.40060

**Published:** 2020-01-01

**Authors:** Lixin Dong, Pei Li, Kaichao Yang, Luming Liu, Hui Gao, Guoyu Zhou, Qian Zhao, Tao Xia, Aiguo Wang, Shun Zhang

**Affiliations:** 1 Department of Occupational and Environmental Health, School of Public Health, Tongji Medical College, Huazhong University of Science and Technology, Wuhan, Hubei, People's Republic of China; 2 Key Laboratory of Environment and Health, Ministry of Education, State Key Laboratory of Environmental health (incubating), School of Public Health, Tongji Medical College, Huazhong University of Science and Technology, Wuhan, Hubei, People's Republic of China; 3 Department of Clinical Nutrition, Tongji Hospital, Tongji Medical College, Huazhong University of Science and Technology, Wuhan, Hubei, People's Republic of China

**Keywords:** PBDE-47, developmental neurotoxicity, mitochondrial fusion and fission, mitochondrial dysfunction, apoptosis.

## Abstract

Polybrominated diphenyl ethers (PBDEs)-induced neurotoxicity is closely associated with mitochondrial abnormalities. Mitochondrial fusion and fission dynamics are required for the maintenance of mitochondrial homeostasis. However, little is known about how PBDEs disrupt this dynamics and whether such disruption contributes to impaired neurodevelopment.

**Methods**: We investigated the effects of 2, 2', 4, 4'-tetrabromodiphenyl ether (PBDE-47), the dominant congener in human samples, on mitochondrial fusion and fission dynamics using PC12 cells, a well-defined *in vitro* neurodevelopmental model. We also evaluated the effects of perinatal low-dose PBDE-47 exposure on hippocampal mitochondrial dynamics and its association with neurobehavioral changes in adult Sprague-Dawley rats.

**Results**: *In vitro*, PBDE-47 disrupted mitochondrial dynamics by inhibiting mitochondrial fusion and fission simultaneously, accompanied by mitochondrial fragmentation, membrane potential dissipation, ATP loss, and apoptosis activation. Specifically, enhancing mitochondrial fusion by the chemical promoter M1 or adenovirus-mediated *mitofusin 2 (Mfn2)* overexpression rescued PBDE-47-caused mitochondrial dynamic, morphological and functional impairments, prevented the resultant apoptosis and promoted neuronal survival. Unexpectedly, either stimulating mitochondrial fission by adenovirus-mediated *fission protein 1 (Fis1)* overexpression or suppressing mitochondrial fission by the mitochondrial division inhibitor-1 (Mdivi-1) failed to reverse whereas aggravated PBDE-47-induced mitochondrial damage and neuronal death. Importantly, promoting mitochondrial fusion by *Mfn2* overexpression neutralized the detrimental effects elicited by *Fis1* overexpression after PBDE-47 treatment. Finally, perinatal oral administration of PBDE-47 elicited neurobehavioral deficits and hippocampal neuronal loss via apoptosis in adult rats, which were associated with mitochondrial dynamics alterations manifested as a fragmented phenotype.

**Conclusion**: Our results suggest that PBDE-47 disrupts mitochondrial dynamics to induce mitochondrial abnormalities, triggering apoptosis and thus contributing to neuronal loss and subsequent neurobehavioral deficits. Targeting mitochondrial fusion may be a promising therapeutic intervention against PBDE-47 neurotoxicity.

## Introduction

Polybrominated diphenyl ethers (PBDEs) are a class of synthetic flame retardants used in many consumer products, including electrical equipment, furniture, textiles, insulation boards and others 1. PBDEs are not chemically bound to the polymers, therefore they can migrate readily from the polymeric materials and enter into the surrounding environment. As emerging persistent organic pollutants, PBDEs have been extensively detected in various environmental and biological samples 2. For the general population, food and dust are major routes of exposure; however, infants and toddlers have additional exposures via placental transfer, breastfeeding and frequent hand-to-mouth behavior 3. In addition to the multiple exposure sources, infants and toddlers have relative lower metabolism and elimination rates, ultimately leading to a higher body burden than in adults 4. This has raised global concerns for the potential developmental toxicity of PBDEs. Specifically, animal studies have demonstrated that exposure to PBDEs during the critical developmental stages causes enduring neurobehavioral problems, especially in motor activity and cognitive domains 5, 6. Additionally, epidemiological data have established the associations between prenatal and/or early-life exposure to PBDEs and the impaired cognitive and behavioral functions in children 2, 7, 8. However, the mechanisms by which PBDEs induce developmental neurotoxicity are still unclear.

Accumulating evidence has suggested that disruption of mitochondrial homeostasis plays a key role in PBDEs neurotoxicity 9. It has been shown that PBDEs are able to preferentially accumulate in mitochondria, suppress mitochondrial oxidative phosphorylation and disturb mitochondrial energy metabolism in cultured neurons and rat brains 10, 11. Moreover, our previous studies have also demonstrated that 2, 2', 4, 4'-tetrabromodiphenyl ether (PBDE-47), the major congener in human biological samples, is capable of impairing mitochondrial morphology and structure, causing mitochondrial membrane potential (MMP) collapse and activating the mitochondrial apoptosis pathway in the developing rat brain and neuronal culture models 12, 13. Nonetheless, how PBDEs elicit neuronal mitochondrial damage is not well understood.

Mitochondria are highly dynamic organelles that frequently undergo fusion and fission to maintain their morphology, size, number and function 14, 15. Fusion is crucial for exchange of mitochondrial contents and helps buffer mild mitochondrial defects as a form of complementation 16. Fission is necessary to produce new mitochondria, contributes to mitophagy (removal of damaged mitochondria) and facilitates apoptosis during severe cellular stress 16. Mitochondrial fusion and fission processes are both mediated by GTPases such as mitofusin 1 (Mfn1), mitofusin 2 (Mfn2), fission protein 1 (Fis1) and dynamin-related protein 1 (Drp1) 15. Mounting evidence suggests that disorders of mitochondrial fusion and fission dynamics have been implicated in various neurological diseases 17, 18. Yet, how PBDE-47 disrupts this dynamics in nervous system and whether such disruption contributes to impaired neurodevelopment remain unknown.

Therefore, in this study, we aimed to investigate the effects of PBDE-47 on neuronal mitochondrial fusion and fission dynamics, and to identify the role of such dynamics disruption in developmental PBDE-47 neurotoxicity. To this end, we used PC12 cells, a well-defined *in vitro* model for neuronal development 19, and an *in vivo* rat model exposed to environmentally relevant levels of PBDE-47 from pre-pregnancy through weaning of offspring to mimic human exposure occurring during the critical developmental periods. We found that PBDE-47 disrupts mitochondrial fusion and fission dynamics to induce mitochondrial abnormalities, resulting in excessive apoptosis and therefore contributing to neuronal loss and subsequent neurobehavioral deficits. We further identified targeting mitochondrial fusion as a potential therapeutic strategy for PBDE-47-induced neurodevelopmental impairments.

## Materials and methods

### Materials

PBDE-47 (purity > 99.99%) was obtained from AccuStandard (New Haven, USA). M1, mitochondrial division inhibitor-1 (Mdivi-1), and dimethyl sulfoxide (DMSO) were purchased from Sigma-Aldrich (St Louis, USA). RPMI 1640 medium was obtained from HyClone (Logan, USA). Fetal bovine serum was purchased from Gibco (carlsbad, USA). Specific primary antibody against caspase-3 was purchased from Cell Signaling Technology (Danvers, USA). Antibodies specific to glyceraldehyde-3-phosphate dehydrogenase (GAPDH), Fis1 and Mfn2, as well as horseradish peroxidase-conjugated anti-rabbit or anti-mouse secondary antibodies were purchased from Proteintech (Wuhan, China). Antibodies specific to Drp1 and Mfn1 were obtained from Abcam (Cambridge, USA). Specific primary antibody against Drp1 phosphorylated at Ser616 was purchased from Signalway Antibody (Baltimore, USA). Cell Counting Kit-8 (CCK-8) and Alexa Fluor 594-conjugated anti-rabbit IgG antibody were purchased from Promoter Biotechnology (Wuhan, China). JC-1 dye, ATP assay kit, BCA assay kit and RIPA lysis buffer were obtained from Beyotime Biotechnology (Shanghai, China). Enhanced chemiluminescence solution was purchased from Advansta (Menlo Park, CA). 3, 3'-diaminobenzidine tetrahydrochloride and MitoTracker Deep Red probe were purchased from Boster Biological Technology (Wuhan, China) and Invitrogen Corp (Carlsbad, CA), respectively.

### Cell culture and treatment

The rat pheochromocytoma PC12 cells were purchased from the Cell Bank of Shanghai Institute of Biochemistry and Cell Biology in Shanghai, China. The cells were grown in RPMI 1640 medium supplemented with 10% (v/v) fetal bovine serum at 37 °C with 5% CO_2_.

The PBDE-47 powder was dissolved in DMSO and diluted to the required concentrations (1.0, 10, or 20 μmol/L) with RPMI 1640 medium before use. PC12 cells, at 70%-80% confluence, were treated with various concentrations of PBDE-47 or DMSO (0.05%) as a vehicle control for 24 h.

To investigate the effects of altered mitochondrial fusion and fission on PBDE-47-induced harmful effects, the cells were treated with PBDE-47 in the presence or absence of mitochondrial fusion promoter M1 (5 μmol/L) or mitochondrial fission inhibitor Mdivi-1 (10 μmol/L, pre-treated for 2 h), or infected with adenovirus expressing *Mfn2* (300 multiplicity of infection (MOI), pre-treated for 24 h, NCBI Reference Sequence: NM_130894.4) or adenovirus expressing *Fis1* (300 MOI, pre-treated for 24 h, NCBI Reference Sequence: NM_001105919.1).

### Cell viability assay

Cell viability was measured by the CCK-8 assay. Cells were planted at a density of 8 × 10^3^ per well in 96-well plates. After treatments, each well was added 10 μL CCK-8 reagent and incubated at 37 °C for 1 h. The absorbance values were obtained at 450 nm by a microplate reader (BioTek Instruments Inc., Winooski, USA). The data were shown as the percentage of control.

### Determination of MMP

MMP was assessed using JC-1 dye. In normal cells, the dye aggregates upon polarization membrane showing orange-red fluorescent. If the MMP dissipates, the dye cannot enter into the transmembrane space, remaining its monomeric form of green. Briefly, the trypsinized cells were centrifuged at 400 g for 5 min, washed with phosphate-buffered saline (PBS), and then incubated with 0.5 mL JC-1 working solution per tube at 37 °C for 30 min. Fluorescent microscopic images of PC12 cells were obtained under an inverted fluorescent microscope (Olympus, Tokyo, Japan) with × 40 objective. In addition, the intensities of red and green fluorescence were also determined by flow cytometry (BD Biosciences, San Jose, USA) at an excitation/emission value of 490/525 nm. The data were expressed as a red/green fluorescence ratio (set to 100% in control).

### ATP measurements

Intracellular ATP levels were determined using an ATP assay kit. After treatment, cells were lysed and centrifuged to collect the cell supernatant. Each well of the black 96-well plate was added and incubated with 100 µL ATP detection working solution for 5 min at room temperature (RT). After that, each well was also added 20 µL cell lysate, and the luminescence was evaluated immediately by a microplate reader (BioTek Instruments Inc., Winooski, USA). The readout was normalized by the protein concentration of each sample.

### Immunofluorescence colocalization

For confocal fluorescence analysis, cells were seeded at a density of 4 × 10^4^ per well in 24-well plates containing polylysine-coated coverslips. After treatment, cells were fixed with 4% paraformaldehyde for 10 min at RT. Following permeabilization with 0.2% Triton X-100, cells were incubated with 100 nmol/L MitoTracker Deep Red probe for 30 min at RT. After blocking with 10% goat serum diluted in PBS for 1 h at RT, cells were stained with active caspase-3 antibody (1:400) at 4 °C overnight, followed by Alexa Fluor 594-conjugated anti-rabbit IgG antibody (green) for 1.5 h at RT. Nuclei were counterstained with 4', 6-diamidino-2-phenylindole (DAPI, blue). Immunofluorescence images were acquired with a laser scanning confocal microscopy (Olympus, Tokyo, Japan).

### Animals and treatment

Sprague-Dawley rats (female and male, weighing 180-220 g) were purchased from the Center for Disease Control and Prevention of Hubei Province, China (certificate No SCXK 2015-0018, Grade specific pathogen free). The rats were maintained under a controlled temperature (22-26 °C) with 50%-60% humidity, 12 h light/dark cycle, fed with standard pellet diet and given tap water ad libitum. All the experimental protocols were approved by the Institutional Animal Care and Use Committee of Tongji Medical College, Huazhong University of Science and Technology, Wuhan, China.

After one-week acclimation to the new environment, female rats were administered PBDE-47 by oral gavage at 0.1, 1.0, 10 mg/kg/day in corn oil or vehicle (corn oil) alone from 10 days before mating, through pregnancy and lactation, until weaning/postnatal day (PND) 21 of offspring. Selection of doses was based on the no-observed-adverse-effect level of 0.7 mg/kg and the lowest-observed-adverse-effect-level of 10.5 mg/kg for developmental PBDE-47 neurotoxicity 20, together with our earlier study that a single oral exposure of PBDE-47 at doses of 1, 5, 10 mg/kg on PND 10 led to neurodevelopmental impairments in adult rats 21. Additionally, comparable doses were also reported for other perinatal low-dose PBDE-47 exposure studies ranging from 0.03 to 1 mg/kg 22-24. Importantly, these doses were well within or slightly above the range reported in environmentally exposed humans 25. The pregnant female rats were housed individually. On day 3 following delivery, the litters were culled to eight pups (four females and four males) to equalize litter size. After weaning, the offspring were divided according to sex and kept in separate cages until PND 88. All animals were sacrificed after the behavioral tests, and the tissues were dissected out immediately. For Nissl staining and immunohistochemical analysis, three brains per group were fixed with 4% paraformaldehyde for 24 h, then embedded in paraffin wax. For ultrastructural observations, the hippocampi (approximately 1 mm cubes) from three brains per group were fixed with 2.5% glutaraldehyde. The other hippocampi were frozen immediately in liquid nitrogen and kept frozen state at -80 °C until use.

### Open field test

Locomotor activity and anxiety were assessed in the open field test. The pups of those litters born within a day of each other (two females and two males per litter) were randomly chosen for the open field test. The test was conducted under dim light and had no unintentional interruptions. The test apparatus, measuring 72 × 72 × 36 cm, was painted black and divided into 25 (5 × 5) equally spaced squares. The "border" was defined as the 16 outer periphery squares of the 25 squares, and the "center" as the 9 squares in the center area. Each rat was placed gently in the center of the apparatus and its behavior was recorded for 5 min. Total distance traveled (cm) and time spent moving (s) were used to assess locomotor activity. The distance traveled and time spent in the central zone were used to evaluate anxiety-like behavior. The floor and walls were cleaned by 10% ethyl alcohol at the end of each trial to eliminate possible bias due to odors left by previous rats.

### Nissl staining

Paraffin-embedded brain samples were sectioned to serial coronal slices (including the hippocampi) with 4 µm thickness, which were subsequently deparaffinized in xylene and stained with cresyl violet (1%) for 5 min. After washing with distilled water, the sections were differentiated in 95% alcohol for 5 min, dehydrated in 95%, 85%, 70% gradient ethanol for 5 min each, cleared with xylene, and covered with neutral balsam and coverslips. Finally, the sections were observed by optical microscope (Olympus, Tokyo, Japan), and the number of Nissl-stained neurons were semi-quantitatively scored by Image-Pro Plus 7.0 software (Media Cybernetics, Silver Spring, USA). For each brain sample, five coronal sections were collected and analyzed.

### Immunohistochemistry

Sections of paraffin-embedded brain samples were deparaffinized in xylene, dehydrated in graded ethyl alcohol and immersed in 3% H_2_O_2_ solution for 10 min to block endogenous peroxidase activity. Subsequently, sections were incubated with 10% normal goat serum in PBS for 1 h to block nonspecific reactions. After washing three times with PBS, the brain sections were incubated overnight with specific primary antibody against Mfn2 (1:200) or Fis1 (1:100) at 4 °C. On the next day, the sections were washed and incubated with anti-rabbit secondary antibody conjugated to horse-radish peroxidase (1:1000) at RT for 30 min. For color development, the sections were treated with 3, 3'-diaminobenzidine tetrahydrochloride for 3 min, and counterstained with hematoxylin. The immunohistochemistry images were acquired by optical microscope (Olympus, Tokyo, Japan). Positive staining was identified and appeared dark brown in cells. The images were also semi-quantitatively analyzed by Image-Pro Plus 7.0 software (Media Cybernetics, Silver Spring, USA). For each brain sample, five coronal sections were collected and analyzed. Values were reported as mean density.

### Transmission electron microscopy (TEM)

PC12 cells and rat hippocampi were first fixed with 2.5% glutaraldehyde at 4 °C overnight and postfixed with 1% osmium tetroxide for another 2 h. After washing with PBS, the samples were dehydrated using graded concentrations of ethanol, infiltrated, embedded in Araldite, stained with 4% uranyl acetate and 0.5% lead citrate, and cut into ultrathin sections (50 nm). The ultrastructure of mitochondria was observed and imaged using a TEM (Philips, Netherlands).

### Western blotting assay

The total proteins of PC12 cells and rat hippocampi were extracted using RIPA lysis buffer. The total protein concentration in samples was measured by a BCA assay kit. The protein samples were separated on sodium dodecyl sulfate-polyacrylamide gels and transferred from the gels onto polyvinylidene fluoride membranes (Roche Applied Science, Indianapolis, IN). After blocking with 5% (w/v) skimmed milk in tris-buffered saline containing 0.1% Tween-20 (TBST) for at least 1 h at RT, the membranes were incubated with appropriate primary antibodies at 4 °C overnight. On the following day, the membranes were washed and incubated with horseradish peroxidase-conjugated anti-rabbit or anti-mouse secondary antibodies for 1 h at RT. After washing three times with TBST, the band intensities were visualized using enhanced chemiluminescence solution, scanned by a GeneGnome chemiluminescent imaging system (Syngene, Cambridge, UK), and quantified with Quantity One software (Bio-Rad, Hercules, CA, USA).

### Statistical analysis

All data are presented as mean ± standard deviation (SD). Data were analyzed using one-way analysis of variance followed by Student-Newman-Keuls post hoc tests for multiple comparisons. Statistical analyses were performed with SPSS software (IBM SPSS, Chicago, IL, USA). *P* value < 0.05 was considered statistically significant.

## Results

### PBDE-47 induces abnormal mitochondrial morphology and function as well as disrupts mitochondrial dynamics in PC12 cells

**Figure 1:**
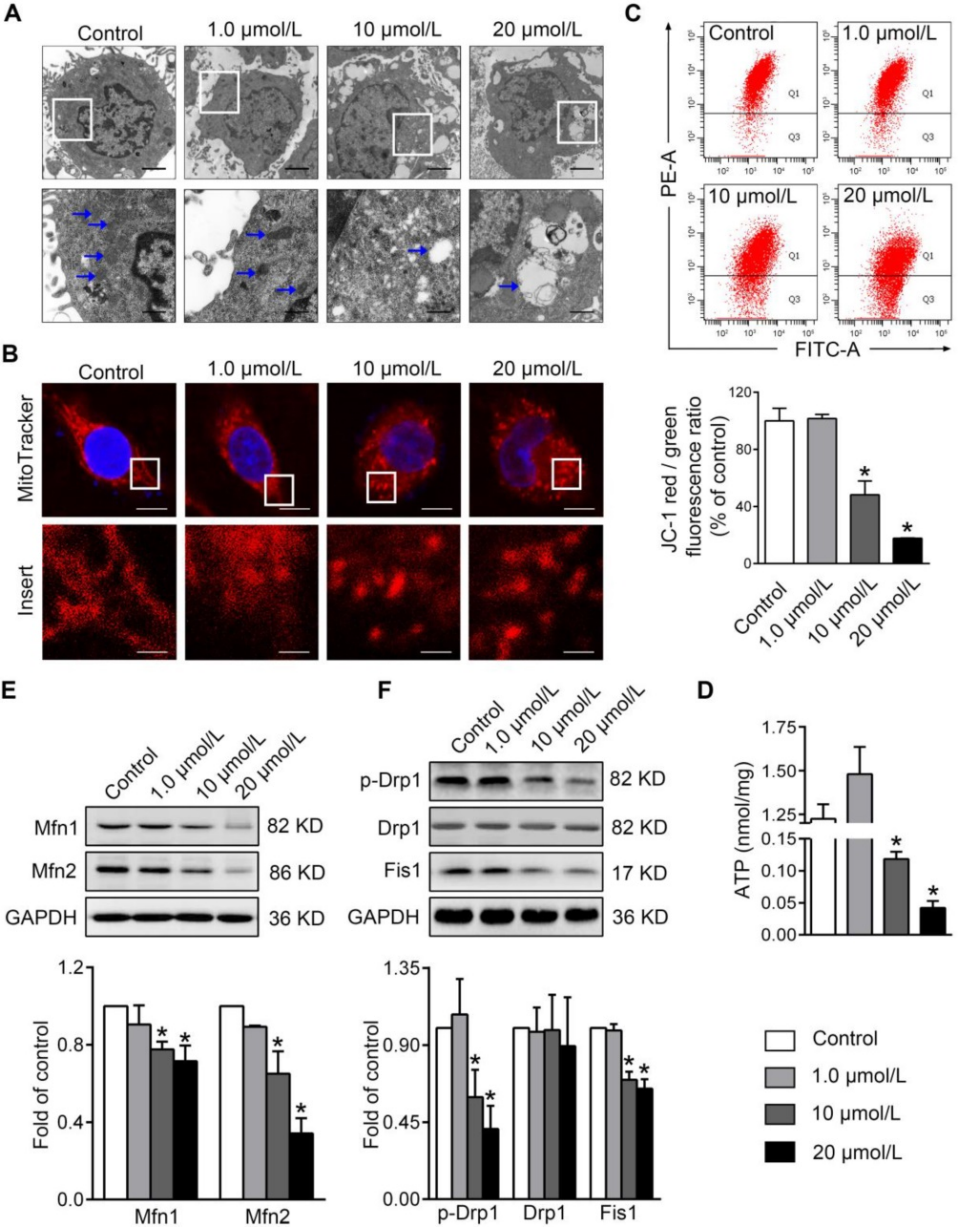
** PBDE-47 induces abnormal mitochondrial morphology and function as well as disturbed mitochondrial dynamics in PC12 cells.** PC12 cells were treated with different concentrations of PBDE-47 (1.0, 10, 20 μmol/L) or DMSO for 24 h. **(A)** Representative TEM images of PC12 cells. Scale bars, 2 μm (top panel), 500 nm (bottom panel); white arrows, mitochondrion. **(B)** Representative confocal images of mitochondrial morphology in PC12 cells. Scale bars, 10 μm (top panel), 2 nm (bottom panel); Red, MitoTracker Deep Red probe staining; blue, DAPI staining. **(C)** Representative flow cytometry plots and quantification of MMP. **(D)** Intracellular ATP levels of PC12 cells. **(E, F)** Representative western blotting and quantification of mitochondrial dynamics proteins. Results are expressed as mean ± SD of three separate experiments. *P < 0.05 versus control group.

To evaluate the impacts of PBDE-47 on mitochondria *in vitro*, we treated PC12 cells with different concentrations of PBDE-47 for 24 h. TEM demonstrated that PBDE-47 treatment induced abnormal mitochondrial ultrastructure manifested as swelling and cristae collapse compared with vehicle control (Figure 1A). Additionally, confocal microscopy revealed that PBDE-47 resulted in a less-elongated and fragmented mitochondrial morphology (Figure 1B). Given mitochondrial morphology are intimately linked to functional states of mitochondria 26, we then determined whether this fragmented mitochondrial phenotype reflects mitochondrial functional changes. Indeed, PBDE-47 reduced the levels of MMP and ATP, two important indicators for mitochondrial function, in a dose-dependent fashion (Figure 1C-D), indicating PBDE-47 induces mitochondrial dysfunction in PC12 cells.

To obtain insights into the molecular basis of the mitochondrial morphological changes by PBDE-47 treatment, we further examined the levels of the major mitochondrial dynamics proteins in this context. Western blotting revealed that PBDE-47 decreased the levels of mitochondrial fusion proteins Mfn1 and Mfn2 in a dose-dependent manner (Figure 1E). A similar alteration in levels of mitochondrial fission proteins Fis1 and phosphorylated Drp1 (p-Drp1 Ser 616, the active form of Drp1) was observed in PBDE-47-treated cells, while no differences in Drp1 levels were detected (Figure 1F). These data suggest that PBDE-47 disrupts mitochondrial dynamics by suppressing fusion and fission simultaneously in PC12 cells.

### Promoting mitochondrial fusion rescues PBDE-47-caused mitochondrial impairments and the resultant neuronal death

**Figure 2:**
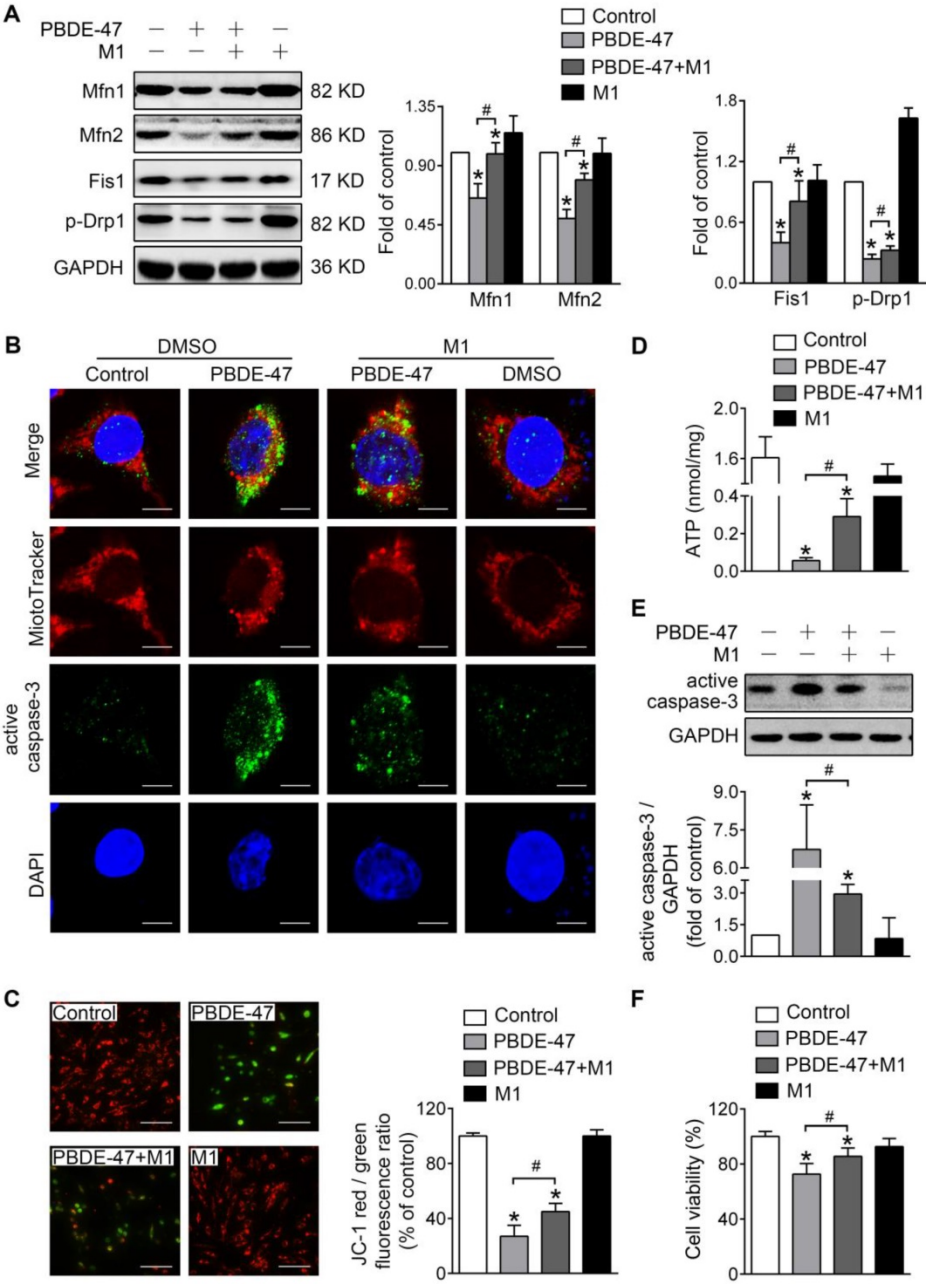
** M1 alleviates PBDE-47-induced mitochondrial impairments and resulting neuronal death.** PC12 cells were treated with PBDE-47 (20 μmol/L) in the presence or absence of mitochondrial fusion promoter M1 (5 μmol/L) for 24 h. **(A)** Representative western blotting and quantification of mitochondrial dynamics proteins. **(B)** Representative confocal images of mitochondrial morphology and caspase-3-positive puncta. Scale bars, 10 μm (top panel), 2 nm (bottom panel); Red, MitoTracker Deep Red probe staining; blue, DAPI staining. **(C)** Representative fluorescent images and quantification of MMP. Scale bar, 100 μm. **(D)** Intracellular ATP levels of PC12 cells. **(E)** Representative western blotting and quantification of active caspase-3. **(F)** Cell viability of PC12 cells. Results are expressed as mean ± SD of three separate experiments. *P < 0.05 versus control group; ^#^*P* < 0.05 versus PBDE-47 group.

To investigate whether mitochondrial fusion suppression plays a role in mitochondrial abnormalities caused by PBDE-47, we first used M1, a potent mitochondrial fusion promoter 27, to enhance mitochondrial fusion. Western blotting analysis showed that M1 significantly mitigated PBDE-47-induced reduction in Mfn1, Mfn2, p-Drp1 and Fis1 levels (Figure 2A). Moreover, M1 alleviated mitochondrial fragmentation and dysfunction induced by PBDE-47 (Figure 2B-D). Since mitochondrial fragmentation is always associated with cell apoptosis 28, we also examined the status of caspase-3, a major downstream executive caspase in apoptosis. Western blotting and confocal immunofluorescence revealed an increased expression of active caspase-3 and an elevation in its colocalization with cells containing fragmented mitochondria after PBDE-47 treatment. However, these effects were blunted by M1 (Figure 2B, E), further correlating mitochondrial fragmentation with PC12 cell apoptosis after PBDE-47 treatment. As expected, the reduced cell viability triggered by PBDE-47 was substantially rescued by M1 (Figure 2F).

**Figure 3:**
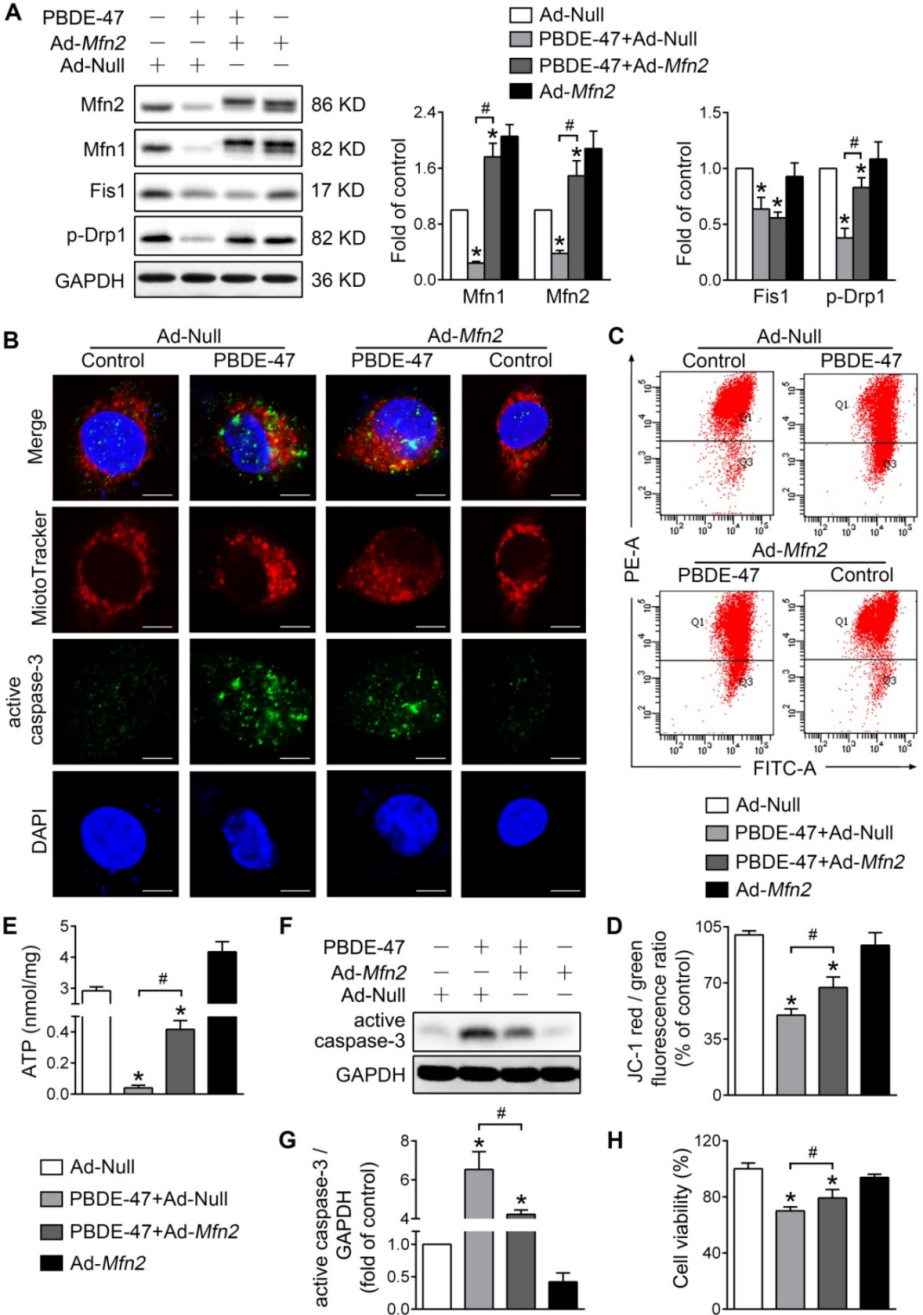
***Mfn2* overexpression alleviates PBDE-47-induced mitochondrial impairments and resulting neuronal death.** After infection with adenovirus expressing *Mfn2* (Ad-*Mfn2*) or control adenovirus (Ad-Null) (MOI=300, pre-treated for 24 h), PC12 cells were treated with PBDE-47 (20 μmol/L) for 24 h. **(A)** Representative western blotting and quantification of mitochondrial dynamics proteins. **(B)** Representative confocal images of mitochondrial morphology and caspase-3-positive puncta. Scale bars, 10 μm (top panel), 2 nm (bottom panel); Red, MitoTracker Deep Red probe staining; blue, DAPI staining. **(C, D)** Representative flow cytometry plots and quantification of MMP. **(E)** Intracellular ATP levels of PC12 cells. **(F, G)** Representative western blotting and quantification of active caspase-3. **(H)** Cell viability of PC12 cells. Results are expressed as mean ± SD of three separate experiments. *P < 0.05 versus control group; ^#^*P* < 0.05 versus PBDE-47 group.

To validate the above results, we also facilitated mitochondrial fusion by adenovirus-mediated *Mfn2* overexpression. Consistent with M1 administration, overexpression of *Mfn2* reversed the reduced Mfn1 and p-Drp1 levels by PBDE-47, although it had no effect on Fis1 level (Figure 3A). In addition, *Mfn2* overexpression attenuated mitochondrial fragmentation and dysfunction caused by PBDE-47 (Figure 3B-E). Especially, overexpression of *Mfn2* blocked PBDE-47-induced cell apoptosis, as evidenced by a decreased expression of active caspase-3 and a concomitant reduction in its colocalization with cells containing fragmented mitochondria (Figure 3B and F-G). Correspondingly, a significant increase in cell viability was observed upon *Mfn2* overexpression after PBDE-47 treatment (Figure 3H). Together, these results suggest that promoting mitochondrial fusion pharmacologically or genetically rescues PBDE-47-induced mitochondrial dynamic, morphological and functional impairments, preventing the resultant apoptosis and thus promoting neuronal survival.

### Either stimulating or suppressing mitochondrial fission fails to rescue whereas aggravates PBDE-47-induced mitochondrial damage and following neuronal death

**Figure 4:**
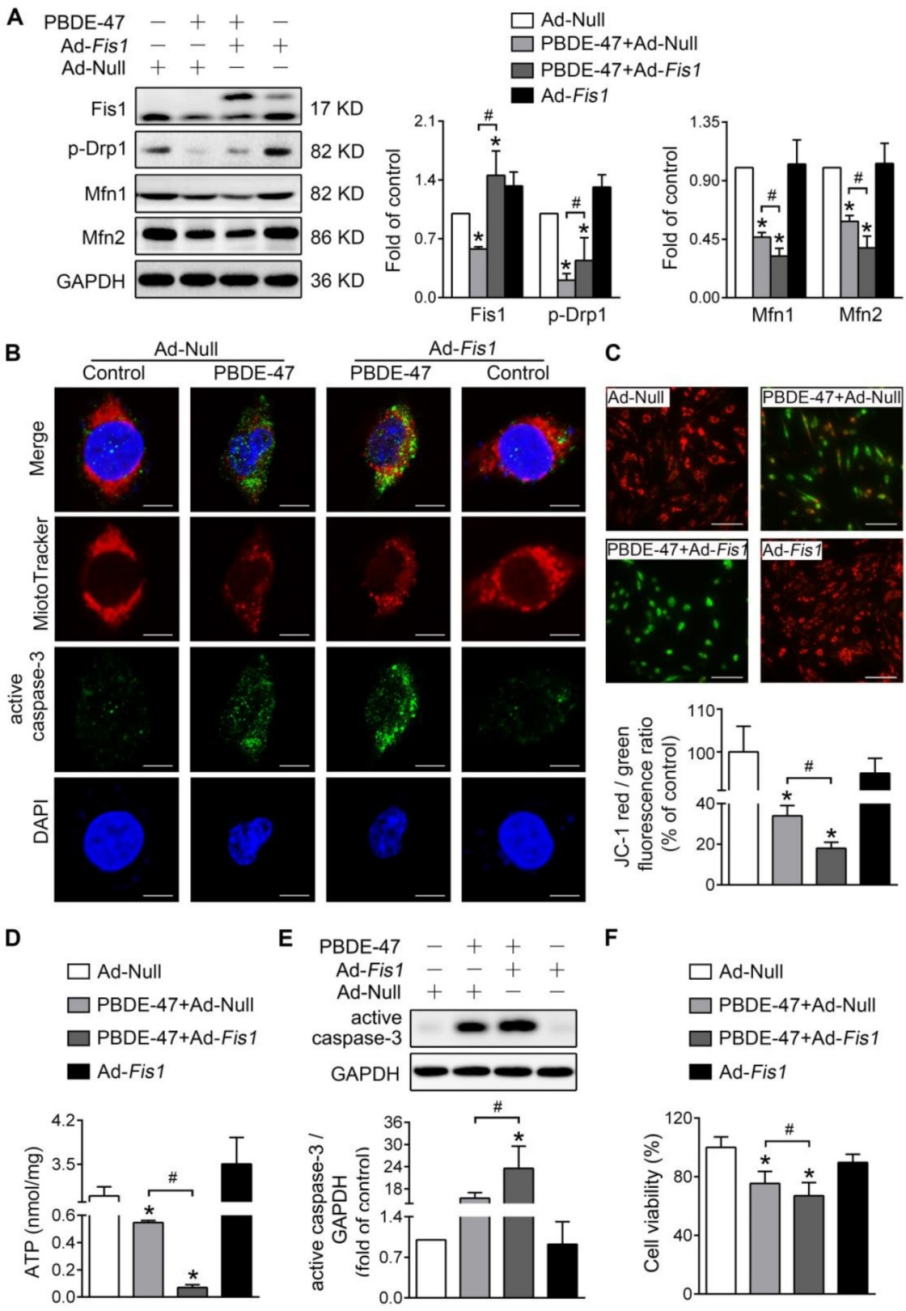
***Fis1* overexpression exacerbates PBDE-47-produced mitochondrial damage and following neuronal death.** After infection with adenovirus expressing *Fis1* (Ad-*Fis1*) or Ad-Null (MOI=300, pre-treated for 24 h), PC12 cells were treated with PBDE-47 (20 μmol/L) for 24 h. **(A)** Representative western blotting and quantification of mitochondrial dynamics proteins. **(B)** Representative confocal images of mitochondrial morphology and caspase-3-positive puncta. Scale bars, 10 μm (top panel), 2 nm (bottom panel); Red, MitoTracker Deep Red probe staining; blue, DAPI staining. **(C)** Representative fluorescent images and quantification of MMP. Scale bar, 100 μm. **(D)** Intracellular ATP levels of PC12 cells. **(E)** Representative western blotting and quantification of active caspase-3.** (F)** Cell viability of PC12 cells. Results are expressed as mean ± SD of three separate experiments. *P < 0.05 versus control group; ^#^*P* < 0.05 versus PBDE-47 group.

Next, we determined whether modulation of mitochondrial fission has the same effects on mitochondrial damage and neuronal death after PBDE-47 treatment. Unexpectedly, stimulating mitochondrial fission by adenovirus-mediated *Fis1* overexpression aggravated PBDE-47-induced inhibition of mitochondrial fusion, manifested as a greater decrease in Mfn1 and Mfn2 levels in spite of partial restoration of p-Drp1 level (Figure 4A). Additionally, overexpression of *Fis1* further potentiated PBDE-47-evoked mitochondrial fragmentation (Figure 4B), mitochondrial dysfunction (Figure 4C-D) and cell apoptosis (Figure 4B, E), along with a more serious decrease in cell viability (Figure 4F).

**Figure 5:**
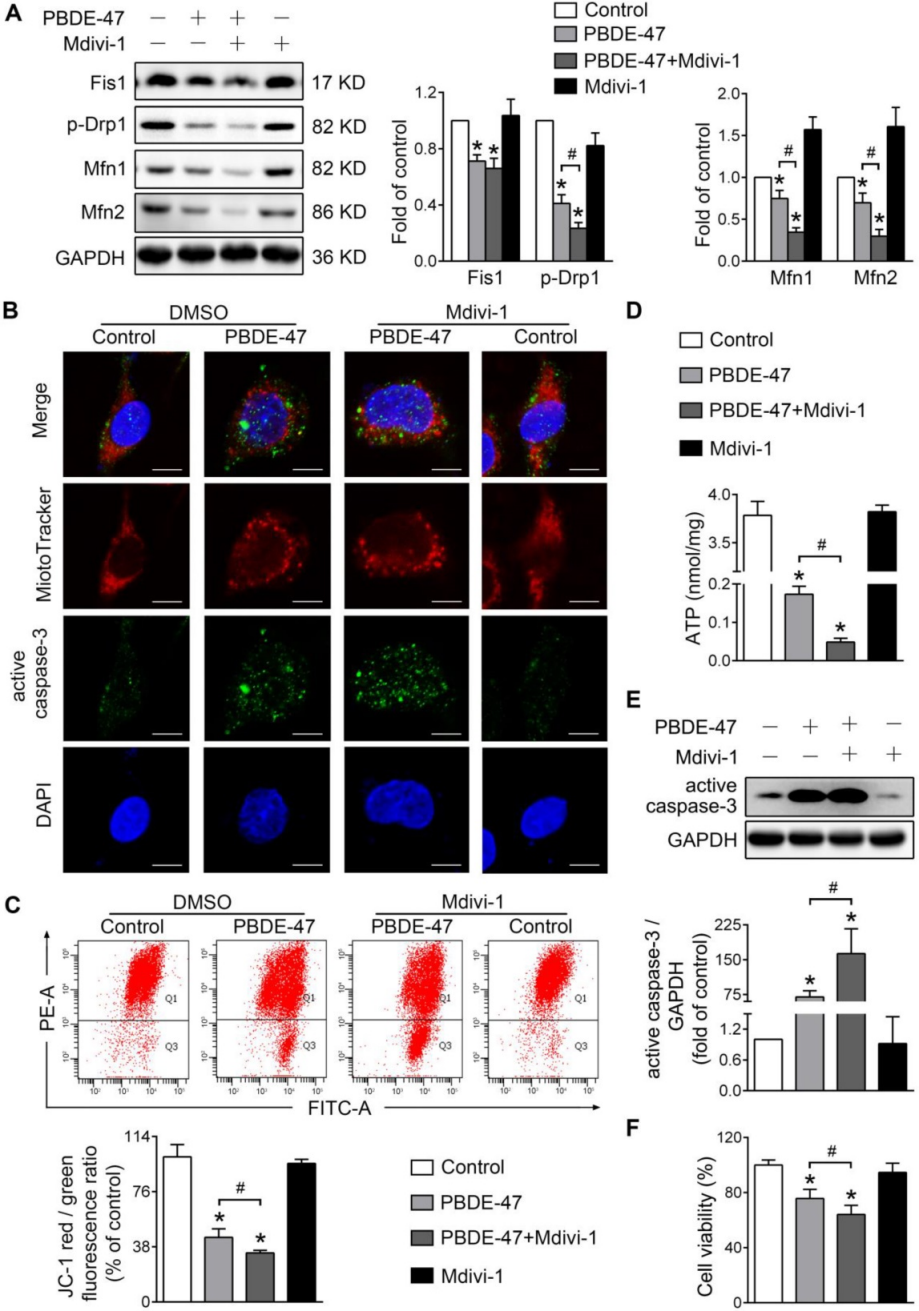
** Mdivi-1 aggravates PBDE-47-produced mitochondrial damage and following neuronal death.** PC12 cells were treated with PBDE-47 (20 μmol/L) in the presence or absence of mitochondrial fission inhibitor Mdivi-1 (10 μmol/L) for 24 h. **(A)** Representative western blotting and quantification of mitochondrial dynamics proteins. **(B)** Representative confocal images of mitochondrial morphology and caspase-3-positive puncta. Scale bars, 10 μm (top panel), 2 nm (bottom panel); Red, MitoTracker Deep Red probe staining; blue, DAPI staining. **(C)** Representative flow cytometry plots and quantification of MMP. **(D)** Intracellular ATP levels of PC12 cells. (**E**) Representative western blotting and quantification of active caspase-3. **(F)** Cell viability of PC12 cells. Results are expressed as mean ± SD of three separate experiments. *P < 0.05 versus control group; ^#^*P* < 0.05 versus PBDE-47 group.

Given the further deteriorative effects of stimulation of mitochondrial fission following PBDE-47 treatment, we then tested whether inhibiting mitochondrial fission exerts a favorable impact under such circumstances 29. Interestingly, Mdivi-1, a selective mitochondrial fission inhibitor, also exacerbated PBDE-47-caused suppression of mitochondrial fusion evidenced by a sustained reduction in Mfn1 and Mfn2 levels (Figure 5A). Similar to the case of *Fis1* overexpression, Mdivi-1 further enhanced PBDE-47-induced mitochondrial fragmentation (Figure 5B), mitochondrial dysfunction (Figure 5C-D) and cell apoptosis (Figure 5B, E). Not surprisingly, Mdivi-1 resulted in an obvious decline in cell viability following PBDE-47 treatment (Figure 5F). Collectively, these results indicate that modulation of mitochondrial fission alone (stimulation or suppression) fails to reverse but promotes PBDE-47-induced mitochondrial dynamic, morphological and functional damage, leading to excessive apoptosis and therefore reducing neuronal survival.

### Promotion of mitochondrial fusion counteracts the harmful effects elicited by stimulation of mitochondrial fission after PBDE-47 treatment

**Figure 6:**
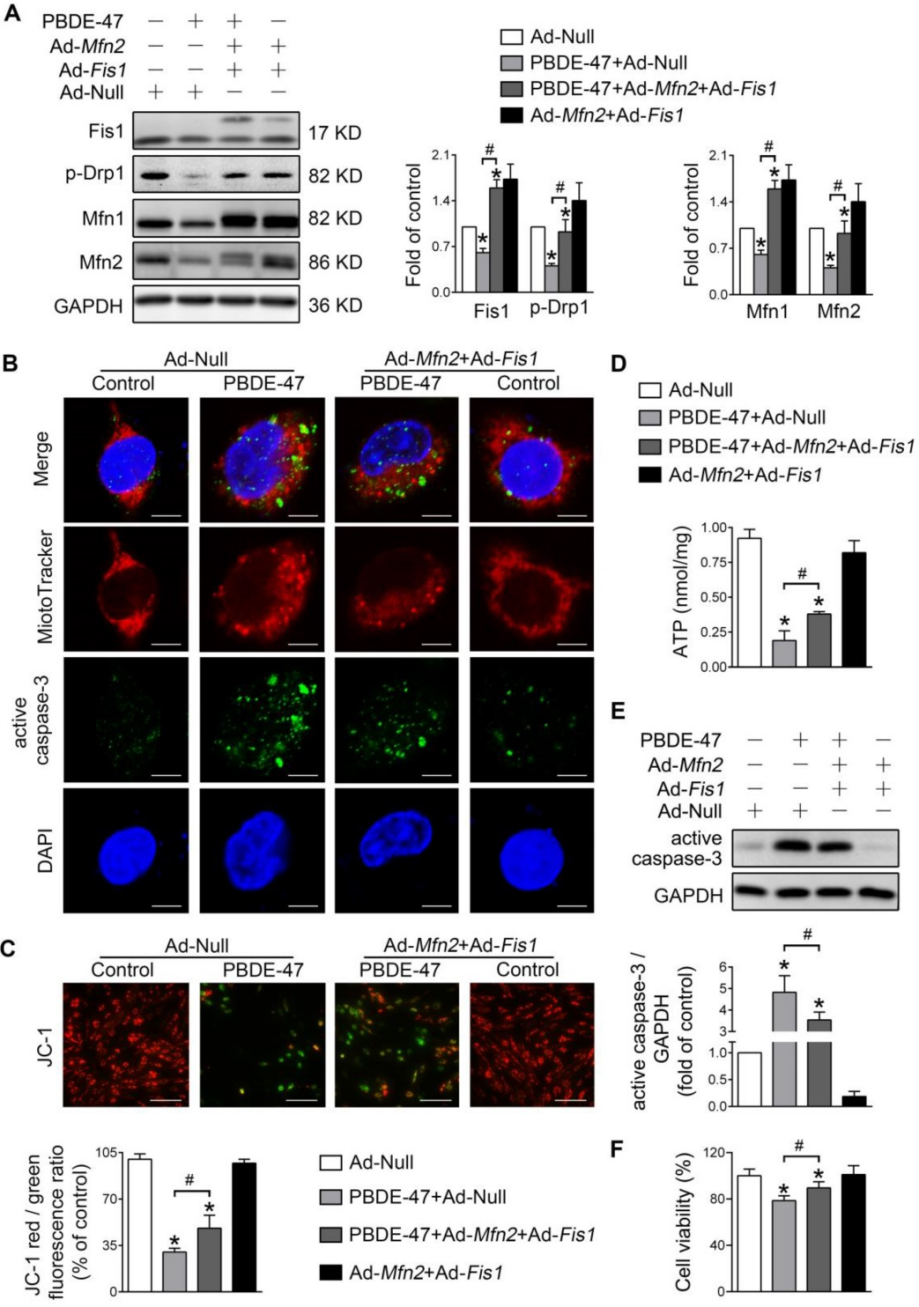
***Mfn2* and* Fis1* double overexpression protects against PBDE-47-induced mitochondrial impairments and resulting neuronal death.** After infection with Ad-*Fis1* plus Ad-*Mfn2* (simultaneous infection) or Ad-Null (all MOI=300, pre-treated for 24 h), PC12 cells were treated with PBDE-47 (20 μmol/L) for 24 h. **(A)** Representative western blotting and quantification of mitochondrial dynamics proteins. **(B)** Representative confocal images of mitochondrial morphology and caspase-3-positive puncta in PC12 cell. Scale bars, 10 μm (top panel), 2 nm (bottom panel); Red, MitoTracker Deep Red probe staining; blue, DAPI staining. **(C)** Representative fluorescent images and quantification of MMP in PC12 cells. Scale bar, 100 μm. **(D)** Intracellular ATP levels of PC12 cells. **(E)** Representative western blotting and quantification of active caspase-3. **(F)** Cell viability of PC12 cells. Results are expressed as mean ± SD of three separate experiments. *P < 0.05 versus control group; ^#^*P* < 0.05 versus PBDE-47 group.

We further explored whether enhancing mitochondrial fusion and fission simultaneously rescues PBDE-47-induced detrimental consequences. Remarkably, double overexpression of* Mfn2* and *Fis1* reversed PBDE-47-induced suppression of mitochondrial fusion and fission, as reflected by a complete restoration of the levels of mitochondrial dynamics proteins (Figure 6A). Moreover, *Mfn2* and* Fis1* double overexpression greatly abolished PBDE-47-induced mitochondrial fragmentation (Figure 6B), mitochondrial dysfunction (Figure 6C-D) and cell apoptosis (Figure 6B, E). Expectedly, the cell viability was significantly elevated upon *Mfn2* and* Fis1* double overexpression after PBDE-47 treatment (Figure 6F). These results collectively suggest that promotion of mitochondrial fusion counteracts the harmful effects elicited by stimulation of mitochondrial fission following PBDE-47 treatment. In this context, simultaneous acceleration of mitochondrial fusion and fission tends toward a normal mitochondrial dynamics, morphology and function, protecting neurons from death via blocking apoptosis.

### Perinatal low-dose PBDE-47 exposure induces neurobehavioral deficits associated with hippocampal mitochondrial dynamics disruption and neuronal loss in adult rats

**Figure 7:**
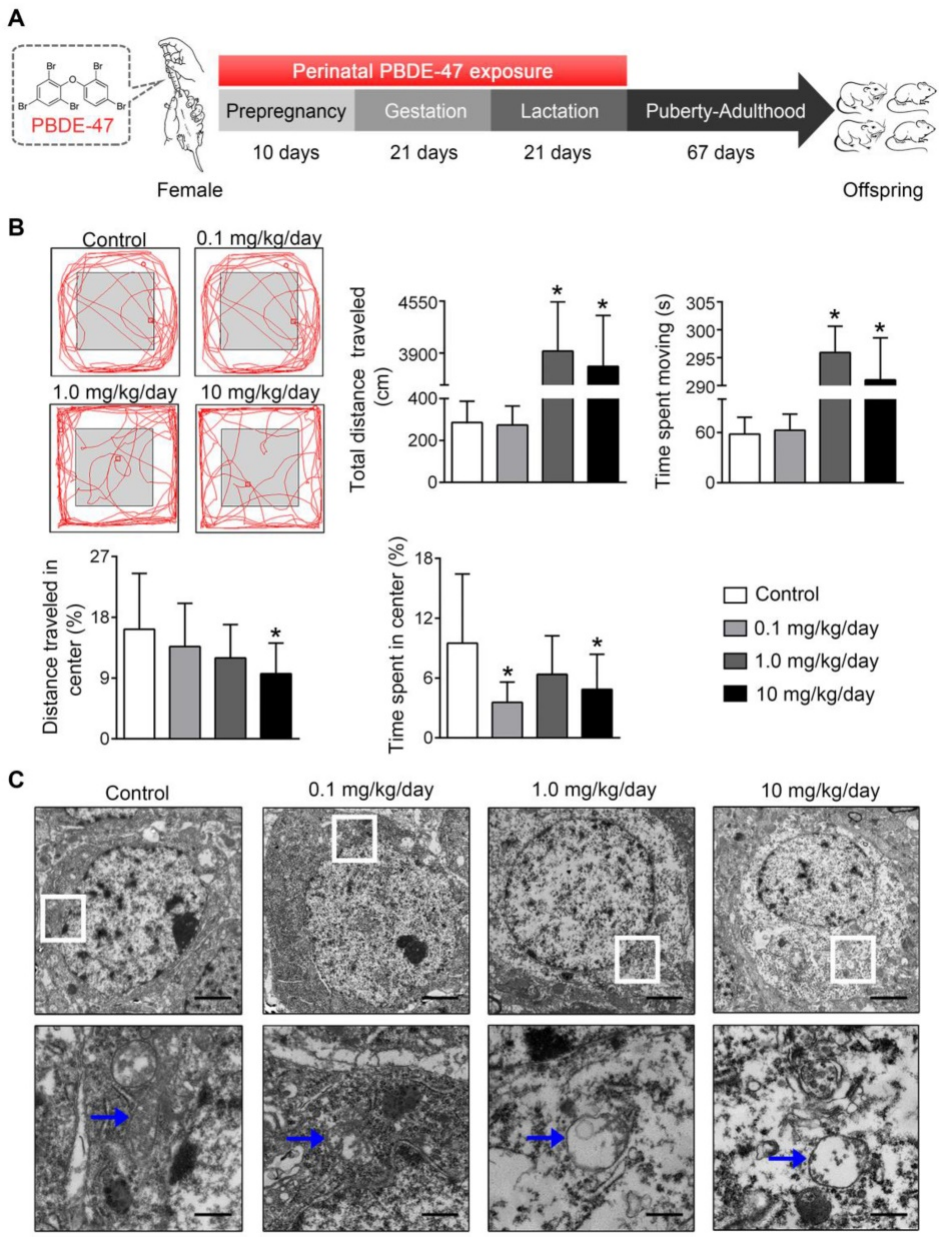
** Perinatal exposure to low doses of PBDE-47 causes neurobehavioral deficits accompanied by hippocampal mitochondrial damage in adult offspring rats. (A)** Schematic of PBDE-47 exposure. **(B)** Representative traces and quantification of the total distance traveled, time spent moving, distance traveled and time spent in the center zone (%) for female rats in the OPT. n=12 rats/group. **(C)** Representative TEM images of hippocampal CA1 region in female rats. n=3 rats/group. Scale bar, 500 μm (Top panel), 50 μm (bottom panel); blue arrows, mitochondrion. Results are expressed as mean ± SD. *P < 0.05 versus control group.

**Figure 8:**
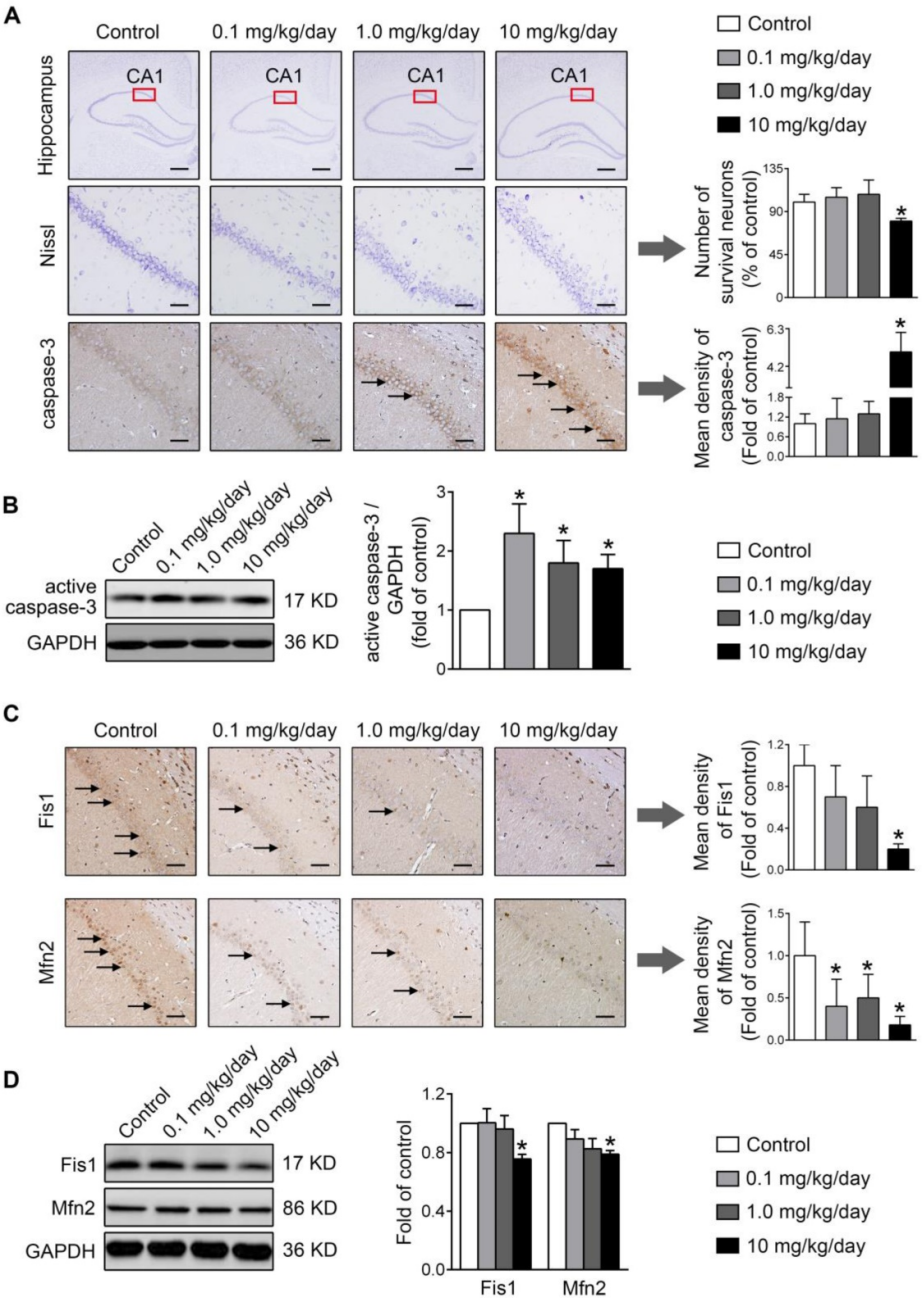
** Perinatal exposure to low doses of PBDE-47 results in hippocampal neuronal apoptosis and loss associated with mitochondrial fusion and fission dynamics disruption in adult female rats. (A)** Representative images and quantification of Nissl staining and caspase-3 immunostaining (black arrows) of hippocampal CA1 region in female rats. n=3 rats/group. Scale bar, 500 μm (top panel), 50 μm (bottom panel). **(B)** Representative western blotting and quantification of active caspase-3 of hippocampus in female rats. n=6 rats/group. **(C)** Representative images and quantification of immunohistochemical staining (black arrows) for Fis1 and Mfn2 of hippocampal CA1 region in female rats. n=3 rats/group. Scale bar, 500 μm. **(D)** Representative western blotting and quantification of Fis1 and Mfn2 of hippocampal tissues in female rats. n=6 rats/group. Results are expressed as mean ± SD. *P < 0.05 versus control group.

Finally, the physiological relevance of the *in vitro* observations was also examined in rats perinatally exposed to environmentally relevant levels of PBDE-47 *in vivo*. The results showed that perinatal oral administration of PBDE-47 caused hyperactivity in adult female rats, manifested as increased total distance traveled and time spent moving in the open field test (Figure 7B). In addition, the distance traveled and time spent in the central zone were decreased, indicating that PBDE-47 exposure increased anxiety levels (Figure 7B). Since spontaneous locomotor activity is closely correlated with neuronal densities and function in hippocampal CA1 region 12, we further assessed whether CA1 neuronal damage is implicated in such behavior deficits. Ultrastructural analysis by TEM revealed that PBDE-47 exposure produced mitochondrial swelling, cristae collapse and fragmentation in hippocampal CA1 neurons (Figure 7C). Moreover, PBDE-47 exposure induced hippocampal CA1 neuronal loss, characterized by a significant reduction of Nissl-stained cells (Figure 8A). This was a consequence of excessive apoptosis as evidenced by the increased expression and intracellular staining of active caspase-3 (Figure 8A-B). Importantly and consistently, perinatal exposure to PBDE-47 also resulted in mitochondrial dynamics disturbance in hippocampi, indicated by simultaneous downregulation of both Mfn2 and Fis1 proteins (Figure 8C-D).

Similar to the effects on female rats, perinatal PBDE-47 exposure induced abnormal spontaneous locomotor activity (Figure S1A-B), hippocampal CA1 mitochondrial damage (Figure S1C) as well as neuronal apoptosis and loss in male rats (Figure S1D-E). These changes were also accompanied by hippocampal mitochondrial dynamics perturbation, manifested as a marked downregulation of p-Drp1 and Fis1 but a notable upregulation of Mfn1 and Mfn2 protein levels (Figure S1F). Overall, these findings indicate that hippocampal mitochondrial dynamics disruption is closely associated with neurobehavioral deficits and neuronal loss in adult rats perinatally exposed to low doses of PBDE-47.

## Discussion

Using a rat model perinatally exposed to environmentally relevant PBDE-47 levels to simulate human exposure happening during critical developmental periods, we showed that such exposure resulted in hyperactivity and anxiety-like behavior in adult rats. Our results are consistent with a previous study where perinatal treatment with PBDE-47 at comparable doses (0.1 and 1.0 mg/kg) caused less distance traveled in the central zone in female mice (PND 60), but it led to a shorter total distance traveled contrary to our finding 22. In addition, another animal study reported that adult mice (PND 70) appeared hypoactive early but hyperactive toward the end in the test period after treatment with a single oral dose of 10.5 mg/kg PBDE-47 on PND 10 30. These inconsistencies may be attributable to differences in exposure and dose regimens, species of laboratory animals and timing of measurements. Most recently, using the same model, we have shown that PBDE-47 exposure also led to memory impairments in adult rats 31. More importantly, these notable impacts of PBDE-47 on neurobehavioral deficits in rodents are wildly supported by the observations of prospective population studies, in which developmental exposure to PBDEs is associated with lower mental and psychomotor development and reduced intelligence quotient at preschool age, or with poorer attention at school age 32. Overall, these findings suggest that PBDE-47 hinders neurodevelopment and corroborate PBDE-47 as a developmental toxicant.

One major observation of this study is that mitochondria exhibited extensive fragmented appearance when both fusion and fission proteins were suppressed in PC12 cells after PBDE-47 treatment. Consistently, perinatal PBDE-47 exposure also decreased the levels of both mitochondrial fusion and fission proteins in hippocampi of female rats. Generally, a decrease in expression of p-Drp1 or Fis1 leads to mitochondrial elongation while reduced expression of Mfn1 or Mfn2 is more typically thought to yield fragmentation 33, 34. However, recent evidence has shown that downregulation of both fusion and fission proteins actually causes mitochondrial fragmentation in neurons 35, 36, which strongly supports our findings. Intriguingly, a little difference was observed in male rats, where such exposure decreased the mitochondrial fusion proteins while increased the fission proteins levels. It is of note that enhanced mitochondrial fission with attenuated mitochondrial fusion also results in fragmentation 37, suggesting that mitochondria display predominantly fragmented changes in the absence of an active fusion process. Indeed, it was the manipulation of fusion but not fission machinery that rescued PBDE-47-induced mitochondrial fragmentation. Therefore, perinatal PBDE-47 exposure virtually produced a similar fragmented mitochondrial phenotype in hippocampi of both female and male rats, despite the sex-specific impacts on mitochondrial fission proteins were observed. Recently, a study demonstrated that PBDE-47 (50 μmol/L) significantly decreased *Mfn2* while increased *Fis1* and *Drp1* mRNA levels in human placental choriocarcinoma BeWo cells 38. Differences in cell-type specificity and dose sensitivity may explain this discrepancy.

Alterations in mitochondrial morphology are known to affect mitochondrial function 39. In agreement with this view, mitochondrial dysfunction was observed in PBDE-47-treated PC12 cells in which mitochondria were fragmented. Hence, it is likely that the elevated mitochondrial fragmentation contributes to PBDE-47-induced mitochondrial dysfunction. Indeed, promoting mitochondrial fusion machinery rescued PBDE-47-caused mitochondrial fragmentation and dysfunction. However, either stimulating or suppressing mitochondrial fission machinery had little effect on PBDE-47-induced those changes in PC12 cells. In particular, the levels of Mfn1 and Mfn2 were decreased further regardless of how mitochondrial fission machinery was manipulated after PBDE-47 treatment. Given Mfn1 and Mfn2 mainly localize to the mitochondria, whereas Drp1 predominantly resides in cytosol and is also involved in other cellular functions including vesicle membrane dynamics 40 and endoplasmic reticulum distribution 41, it is possible that mitochondrial fusion is dominant over fission for the regulation of mitochondrial morphology and function following PBDE-47 treatment. More importantly, such mitochondrial abnormalities were largely restored via simultaneous acceleration of fusion and fission machineries, indicating that mitochondrial fusion neutralizes the detrimental effects elicited by mitochondrial fission perturbation after PBDE-47 treatment. Taken together, these results suggest that mitochondrial fusion is indispensable for the maintenance of organelle fidelity in PBDE-47-treated neurons, in keeping with the notion that mitochondrial fusion is crucial for maintaining MMP and respiratory capacity in mammalian cells 42-44.

The current study, together with our recent work 31, demonstrated that perinatal PBDE-47 exposure led to hippocampal CA1 and CA3 neuronal loss attributable to excessive apoptosis, which was confirmed in PC12 cells. It is known that crippling mitochondrial fusion sensitizes cells to apoptotic stimuli 45 and mitochondrial fragmentation is an early event contributing to cell apoptosis 46. In line with this, promoting mitochondrial fusion machinery prevented PBDE-47-induced mitochondrial fragmentation, caspase-3 activation and PC12 cell death. In contrast to modulating mitochondrial fission machinery alone, simultaneous acceleration of mitochondrial fusion and fission machineries was moved toward a more balanced state that was beneficial for cell survival. The present study further extended our previous findings 12, 13 by showing that mitochondrial dynamics disruption was also implicated in PBDE-47-elicited neuronal loss via inducing excessive apoptosis. Altogether, these findings suggest that manipulation of mitochondrial fusion machinery is able to balance mitochondrial dynamics, restore mitochondrial morphology and function, inhibit excessive apoptosis, ultimately improving neuronal survival after PBDE-47 exposure.

**Figure 9:**
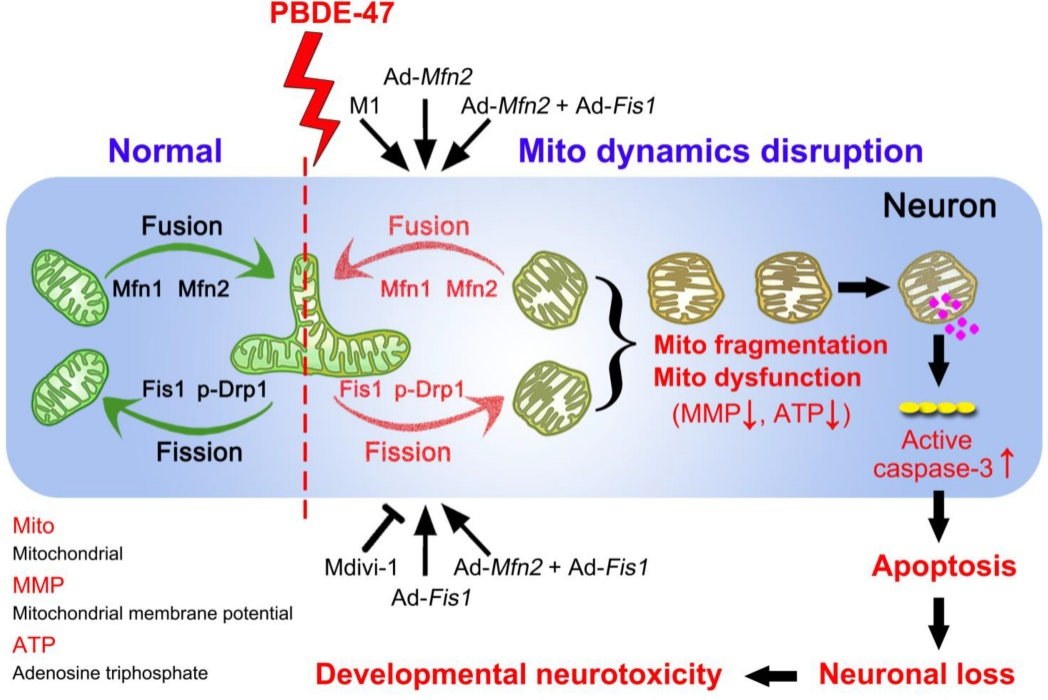
** Schematic representation for the role of mitochondrial dynamics disruption in PBDE-47-caused developmental neurotoxicity.** PBDE-47 induced mitochondrial fragmentation and dysfunction (MMP dissipation and ATP loss) by disrupting mitochondrial fusion and fission dynamics, resulting in excessive apoptosis and thus contributing to neuronal death *in vivo* and *in vitro*. Specifically, enhancing mitochondrial fusion by the chemical promoter M1 or adenovirus-mediated *Mfn2* overexpression alleviated PBDE-47-caused mitochondrial morphological and functional impairments, prevented the resultant apoptosis and promoted neuronal survival. Unexpectedly, either stimulating mitochondrial fission by adenovirus-mediated *Fis1* overexpression or suppressing mitochondrial fission by the division inhibitor Mdivi-1 failed to rescue, whereas aggravated PBDE-47-induced mitochondrial damage and neuronal death. More importantly, promoting mitochondrial fusion by *Mfn2* overexpression reversed the detrimental effects elicited by *Fis1* overexpression following PBDE-47 treatment.

In summary, we demonstrate here that disruption in mitochondrial fusion and fission dynamics contributes to PBDE-47-elicited developmental neurotoxicity. Particularly, we have uncovered that this dynamics perturbation induces mitochondrial fragmentation and dysfunction, triggering excessive apoptosis and thus resulting in neuronal loss. More importantly, we have identified that promotion of mitochondrial fusion protects against PBDE-47-elicited detrimental effects in contrast to modulation of mitochondrial fission machinery (Figure 9). Our results provide novel insights into the molecular mechanisms by which neurons respond to PBDE-47 action and underscore the importance of targeting mitochondrial fusion as a potential therapeutic strategy for PBDE-47 neurotoxicity.

## Supplementary Material

Supplementary figure.

## Abbreviations

CCK-8: Cell Counting Kit-8
DAPI: 4', 6-diamidino-2-phenylindole
DMSO: dimethyl sulfoxide
Drp1: dynamin-related protein 1
Fis1: fission protein 1
GAPDH: glyceraldehyde-3-phosphate dehydrogenase
Mdivi-1: mitochondrial division inhibitor-1
Mfn1: mitofusin 1
Mfn2: mitofusin 2
MMP: mitochondrial membrane potential
MOI: multiplicity of infection
PBDE-47: 2, 2', 4, 4'-tetrabromodiphenyl ether
PBDEs: polybrominated diphenyl ethers
PBS: phosphate-buffered saline
PND: postnatal day
RT: room temperature
SD: standard deviation
TBST: tris-buffered saline containing 0.1% Tween-20
TEM: transmission electron microscopy
