# Supplementary material for: Promotion of mitochondrial fusion protects against developmental PBDE-47 neurotoxicity by restoring mitochondrial homeostasis and suppressing excessive apoptosis
Source: Theranostics. 2020 Jan 1;10(3):1245–61. doi: 10.7150/thno.40060 (PMC6956817; doi:10.7150/thno.40060)
Supplement: Supplementary file 1 — Supplementary figure. [file thnov10p1245s1.pdf]

Supplementary Material

Promotion of mitochondrial fusion protects against developmental PBDE-47 neurotoxicity by restoring mitochondrial homeostasis and suppressing excessive apoptosis

Lixin Dong [a,b, 1], Pei Li [a,b, 1], Kaichao Yang [a,b,], Luming Liu [a,b], Hui Gao [c], Guoyu Zhou [a,b], Qian Zhao [a,b], Tao Xia [a,b], Aiguo Wang [a,b], Shun Zhang [a,b,]*

[a] Department of Occupational and Environmental Health, School of Public Health, Tongji Medical College, Huazhong University of Science and Technology, Wuhan, Hubei, People's Republic of China

[b] Key Laboratory of Environment and Health, Ministry of Education, State Key Laboratory of Environmental health (incubating), School of Public Health, Tongji Medical College, Huazhong University of Science and Technology, Wuhan, Hubei, People's Republic of China

[c] Department of Clinical Nutrition, Tongji Hospital, Tongji Medical College, Huazhong University of Science and Technology, Wuhan, Hubei, People's Republic of China

[1] These authors contributed equally to this work.

***Corresponding author:**

Dr. Shun Zhang, Phone: 86-27-83691030, Fax: 86-27-83692701,

Email: shunzhang@hust.edu.cn

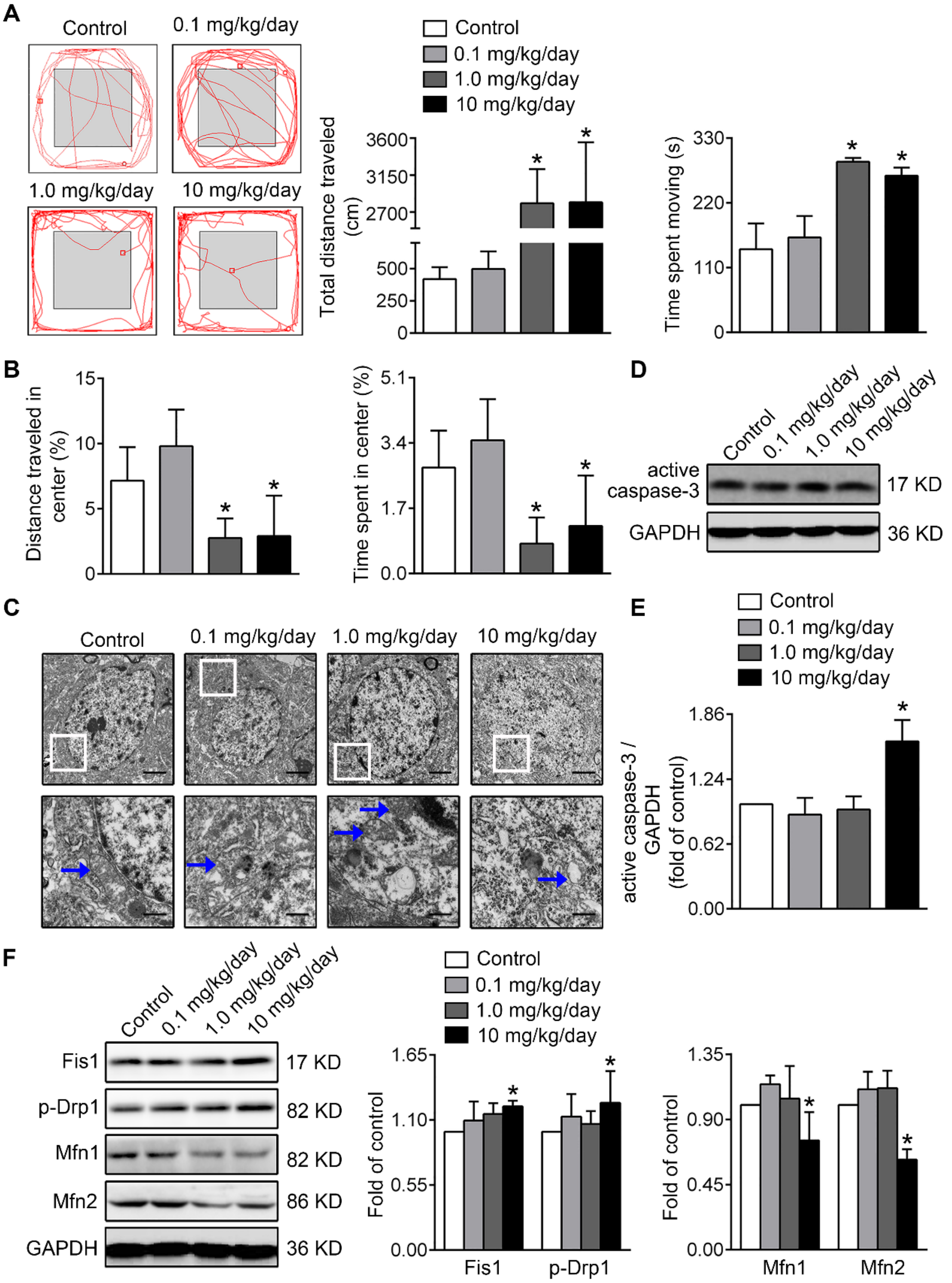

Figure S1 Perinatal low-dose PBDE-47 exposure induces hippocampal mitochondrial fusion and fission disequilibrium associated with neuronal apoptosis in male offspring rats. (**A, B**) Representative traces and the quantification of the total distance traveled, time spent moving, distance traveled and time spent in the central zone (%) for male

rats in the OPT. n=12 rats/group. (**C**) Representative TEM images of hippocampal CA1 region in male rats. n=3 rats/group. Scale bar, 500 μm (top panel), 50 μm (bottom panel); blue arrows, mitochondria. (**D-F**) Representative western blotting and quantification of active caspase-3 (**D, E**) as well as mitochondrial dynamics proteins (**F**) of hippocampal tissues in male rats. n=6 rats/group. Results are expressed as mean ± SD. *$P < 0.05$ versus control group.
